# Frontal Functional Network Disruption Associated with Amyotrophic Lateral Sclerosis: An fNIRS-Based Minimum Spanning Tree Analysis

**DOI:** 10.3389/fnins.2020.613990

**Published:** 2020-12-23

**Authors:** Seyyed Bahram Borgheai, John McLinden, Kunal Mankodiya, Yalda Shahriari

**Affiliations:** ^1^Department of Electrical, Computer, and Biomedical Engineering, University of Rhode Island, Kingston, RI, United States; ^2^Interdisciplinary Neuroscience Program, University of Rhode Island, Kingston, RI, United States

**Keywords:** MST, graph theory, functional connectivity, PLV, fNIRS, ALS, executive dysfunction

## Abstract

Recent evidence increasingly associates network disruption in brain organization with multiple neurodegenerative diseases, including amyotrophic lateral sclerosis (ALS), a rare terminal disease. However, the comparability of brain network characteristics across different studies remains a challenge for conventional graph theoretical methods. One suggested method to address this issue is minimum spanning tree (MST) analysis, which provides a less biased comparison. Here, we assessed the novel application of MST network analysis to hemodynamic responses recorded by functional near-infrared spectroscopy (fNIRS) neuroimaging modality, during an activity-based paradigm to investigate hypothetical disruptions in frontal functional brain network topology as a marker of the executive dysfunction, one of the most prevalent cognitive deficit reported across ALS studies. We analyzed data recorded from nine participants with ALS and ten age-matched healthy controls by first estimating functional connectivity, using phase-locking value (PLV) analysis, and then constructing the corresponding individual and group MSTs. Our results showed significant between-group differences in several MST topological properties, including leaf fraction, maximum degree, diameter, eccentricity, and degree divergence. We further observed a global shift toward more centralized frontal network organizations in the ALS group, interpreted as a more random or dysregulated network in this cohort. Moreover, the similarity analysis demonstrated marginally significantly increased overlap in the individual MSTs from the control group, implying a reference network with lower topological variation in the healthy cohort. Our nodal analysis characterized the main local hubs in healthy controls as distributed more evenly over the frontal cortex, with slightly higher occurrence in the left prefrontal cortex (PFC), while in the ALS group, the most frequent hubs were asymmetrical, observed primarily in the right prefrontal cortex. Furthermore, it was demonstrated that the global PLV (gPLV) synchronization metric is associated with disease progression, and a few topological properties, including leaf fraction and tree hierarchy, are linked to disease duration. These results suggest that dysregulation, centralization, and asymmetry of the hemodynamic-based frontal functional network during activity are potential neuro-topological markers of ALS pathogenesis. Our findings can possibly support new bedside assessments of the functional status of ALS’ brain network and could hypothetically extend to applications in other neurodegenerative diseases.

## Introduction

In recent years, there has been growing interest in associations between disruptions in brain network topology and a range of neurodegenerative diseases (Stam et al., 2009; Stam, 2014; McMackin et al., 2019), including amyotrophic lateral sclerosis (ALS) (Zhou et al., 2016; Sorrentino et al., 2018), a rare terminal neurodegenerative condition generally characterized by progressive deficits in motor neurons. This tendency stems from a conceptual shift from a reductionist view of brain organization as a mere sum of independent constituent regions toward a more integrative network view, which has been propelled to applying network science in neuroimaging studies (Bullmore and Sporns, 2012; Olde Dubbelink et al., 2014). Particularly for ALS, this interest grows out of a change in our perception of the disease from a mere motor system pathology to a multifaceted disease, which includes non-motor disruptions involving behavioral and cognitive functions (Beeldman et al., 2016; Kellmeyer et al., 2018). Up to 50% of people with ALS are reported to have cognitive impairments and behavioral disorders, which include frontotemporal lobar degeneration (FTLD), affecting 15% to 41% of patients (Lomen-Hoerth et al., 2003). Among cognitive impairments, executive dysfunction is the most prevalent deficit, affecting about 40% of non-demented ALS patients (Lomen-Hoerth et al., 2003; Beeldman et al., 2016). Montuschi et al. (2015) report that 13% of ALS patients they assessed displayed symptoms of dementia, while 37% demonstrated non-demented executive impairments. Executive dysfunctions in ALS patients are typically associated with impairments in verbal fluency, working memory (WM) processing, dual tasking functions, sustained and selective attention, cognitive inhibition, and visual attention (Pettit et al., 2013; Murphy et al., 2016). As executive dysfunctions are generally related to deficits in the frontal cortex (Alvarez and Emory, 2006), recognizing the underlying structural and functional neurocorrelates of these type of impairments in the frontal regions of the cortex would advance our understanding of the pathological and prognostic patterns of the disease and lead to more efficient diagnostic and treatment methods.

Although advanced non-invasive neuroimaging methods have recently been proposed to fulfill this aim, little understanding has been gained about the cortical organizations underlying executive dysfunction in ALS patients. A common finding of structural changes in the non-motor cortical regions of ALS patients is a reduction in frontal and prefrontal white matter density (Douaud et al., 2011; Agosta et al., 2013; Dimond et al., 2017). Notably, structural and functional connectivity degeneration are reported to be coupled in these cohorts and mutually affected by the degeneration associated with the disease. Accordingly, there is a rising interest in determining the functional connectivity underpinning the pathogenesis of ALS. Several studies have adopted resting-state functional connectivity (RSFC) to characterize potential neurophysiological biomarkers of ALS. However, divergent outcomes have hindered a clear consensus on both the functional connectivity markers and their proper interpretations in ALS (Luo et al., 2012; Schmidt et al., 2014; Fraschini et al., 2018). Therefore, there is a strong need for complementary approaches to improve our understanding of network alterations underlying disease-related dysfunctions, particularly frontal markers in the presence of non-motor impairments.

One shortcoming of connectivity studies is that they primarily focus on the comparison of individual connections and thus are inadequate in providing a global and integrative perspective on the brain network (Jonak et al., 2019). Graph theory has recently shown promise in compensating for this shortfall and bridging between network disruptions and neurodegenerative diseases (Stam et al., 2009; Stam, 2014) by modeling the brain as a whole network with recording channels, or regions of interest, as nodes and their functional interaction, or structural interconnections, as links. By employing integrative network metrics, these methods provide a ground to reconstruct and compare the global and local characteristics of the brain network’s organizations between different groups and experimental conditions. With regard to ALS, a handful of studies (Verstraete et al., 2014; Buchanan et al., 2015) have adopted graph-theoretical methods to address structural, but not functional, disruptions in patients’ brain networks by constructing the brain network in terms of the interlinking white matter tracts between segmented regions. Notably, these studies have commonly reported a loss of local connectivity in the motor area, which expands to the frontal and parietal regions.

However, a remaining challenge for conventional graph-theoretical methods is the limited comparability between brain network characteristics across groups or conditions in different studies, as they involve arbitrary choices in the normalization stage. These arbitrary choices project an intrinsic bias to these methods, and thus, lead to inconsistent findings across different studies (van Wijk et al., 2010; Tewarie et al., 2015). One suggested method to compensate for this problem is the minimum spanning tree (MST) analysis, which avoids methodological biases. The MST network is a sub-graph that traverses all nodes by minimizing the cost (link weights) without forming a loop (Tewarie et al., 2015; van Dellen et al., 2018). Link weights in neuroimaging studies are typically attributed in terms of functional connectivity measures. The MST is, in principle, not sensitive to scaling effects, as its structure depends merely on the order rather than the absolute values of link weights (Jackson and Read, 2010). Although in converting a fully connected weighted network to the unweighted MST, we may lose some information, it has been frequently shown that the MST sub-network can preserve essential network properties (King and Tidor, 2009; Tewarie et al., 2015) and be equally sensitive to topological alterations as conventional network analysis methods, such as clustering and path length (Tewarie et al., 2015).

Initiated by Lee et al. (2006) to characterize patients with temporal epilepsy, MST analysis has been recently adopted in an increasing number of neuroimaging studies (Olde Dubbelink et al., 2014; Fraga González et al., 2016; Li et al., 2017; Jonak et al., 2019). As an MST subnetwork is derived from a weighted connectivity network, it is insensitive to the nature of the connectivity measure or the imaging modality. This offers great potential for the method to be employed in a variety of neuroimaging studies investigating different populations and conditions. However, only a few studies have used this method to gain a better understanding of the underlying disruptions in the brain networks of ALS patients. In an electroencephalography (EEG) resting-state network study, Fraschini et al. (2016) report significant differences in the topological properties of MSTs constructed over functional connectivity matrices of ALS patients compared to controls. They have also observed significant correlations between network metrics and disability scores in patients. In another resting-state magnetic resonance imaging (MRI)-magnetoencephalography (MEG) study using MST analysis, Sorrentino et al. (2018), report more connected and scale-free brain networks as the disease develop in ALS patients.

However, in addition to being limited in numbers, there are some concerns about expanding the topological findings reported in these few studies as neurological markers associated with the prognosis and progress of the disease, particularly in the presence of non-motor and executive dysfunctions. First, these studies, like the majority of works adopting network and connectivity analysis, are focused only on resting-state experimental paradigms, which lack the potential to identify disruption of brain network organizations while subjects actively perform tasks. Although resting-state paradigms are advocated to be insensitive to performance variability across subjects (Luo et al., 2012) and, accordingly, a better candidate for structural impairments, they cannot completely mirror the inter-regional functional deficits underlying the disease during daily life activities. In particular, when it comes to addressing the cognitive and executive dysfunctions associated with motor impairments in ALS patients, there is a strong need to investigate topological disruptions during a cognitive task. Second, non-portable and bulky neuroimaging systems such as MRI or MEG are not compatible with the specific physical conditions of ALS patients, particularly as the disease progresses and the patients lose their mobility. Therefore, there is an increasing interest in more portable and flexible neuroimaging equipment for use at patients’ bedsides (Linden and Fallgatter, 2009; Kellmeyer et al., 2018). EEG is generally a suitable candidate to fulfill this goal with its high temporal resolution, which provides the foundation for functional connectivity analysis in different frequency bands (Kellmeyer et al., 2018; Bareham et al., 2020). However, due to its relatively low spatial resolution and its low signal-to-noise ratio (SNR), likely due to its high sensitivity to artifacts, it is not competitive, especially when the region of interest includes the prefrontal and anterior frontal channels that can include artifacts from eye-blink and forehead muscle movement. With regard to ALS, this modality does not prove to offer an adequate competitive advantage when the aim of the study is to address executive dysfunction in the frontal lobe. For instance, in the aforementioned MST-based EEG study pursued by Fraschini et al. (2016), several prefrontal channels were excluded from further analysis due to probable contamination from muscular or ocular artifacts. Therefore, more suitable portable neuroimaging methods are required to capture network disruptions associated with executive dysfunction in the ALS cohort. Functional near-infrared spectroscopy (fNIRS) has been recently introduced as a non-invasive portable neuroimaging system to mirror hemodynamic perturbations in a range of neurodegenerative diseases, including ALS (Maidan et al., 2016; Kahya et al., 2019; Borgheai et al., 2020). Compared to EEG, fNIRS systems provide higher spatial resolution and lower sensitivity to artifacts. In connectivity analysis, fNIRS nullifies spurious inter-regional functional relations and thus is less affected by volume conductance (Molavi et al., 2014). Interestingly, fNIRS systems have shown promising results in mirroring alterations in cerebral oxygenation in response to the activation of the prefrontal and frontal cortices through mental tasks such as mental arithmetic operations (Bauernfeind et al., 2011; Schudlo and Chau, 2014). Although an increasing number of studies have employed resting-state fNIRS connectivity analysis to characterize neurodegenerative diseases (either channel-wise or clustered using graph theory) (Li et al., 2018; Niu and Zhu, 2019), few have been conducted to investigate connectivity or network patterns in an active paradigm while subjects perform a task. Moreover, to the best of our knowledge, no fNIRS study has adopted MST network analysis to address topological properties in the brain network.

In this study, to capture the frontal topological disruptions associated with executive dysfunctions in ALS patients, for the first time, we have adopted MST analysis to map the neuroimaging data recorded through fNIRS during a proposed visuo-mental paradigm. Following our previous work that shows frontal channel-wise differences over the first order hemodynamic properties in ALS patients during the same proposed task (Borgheai et al., 2019), in the present study, we hypothesize that there are probable network disruptions related to executive dysfunctions in ALS patients reflected in MST graphs constructed over hemodynamic-based functional connectivity metrics. We will also demonstrate that applying MST mapping to fNIRS-based hemodynamic signals through a cognitive task is a feasible and fruitful process. We will further investigate whether the global properties gained through network analysis are associated with the clinical records of ALS patients. The outcomes from this study can extend further to develop bedside fNIRS-based systems to explore neuro-topological markers of disease pathogenesis and prognosis.

## Materials and Methods

### Participants

Nineteen participants were recruited for this study and were divided into two groups: 9 individuals diagnosed with ALS (age: 58.2 ± 12.9, seven males) and 10 age-matched healthy controls (HC) (age: 60.5 ± 11.6, four males). The demographic and clinical information of the ALS group, including age, sex, disease duration, disability score, and education level, are listed in Table 1. Revised ALS functional rating scale (ALSFRS-R) scores, a validated screen for the dysfunctional progression of the ALS disease, averaged 19.2 ± 15.0 on a 48-point scale, where the highest score (48) reflects normal function in activities of daily living (ADL), and the lowest score (0) represents a complete loss of function (Cedarbaum et al., 1999). Their disease durations were 4.9 ± 4.0 years on average. Three patients (ALS-1, 2, and 4) had gastrostomies as well as tracheostomies. All participants in both groups had at least some post-secondary education. Healthy controls acknowledged no history of visual, mental, or substance-related disorders. One of the healthy controls showed insufficient channel quality in the calibration stage and was, therefore, excluded from further analysis. All procedures were in accordance with the study protocol approved by the Institutional Review Board (IRB) of the University of Rhode Island (URI). All participants provided informed consent or assent prior to the experiment and were financially compensated. All participants in the ALS group were tested in either their homes or care centers, while the healthy cohort participated in the experiments in the NeuralPC lab at URI.

**TABLE 1:** ALS participants’ demographic information.

*Subject no.*	*Age*	*Sex*	*Disease duration (years)*	*ALSFRS-R (max 48)*	*Education level*
ALS-1	29	M	4	0	College degree
ALS-2	55	M	11	4	Graduate degree
ALS-3	70	M	8	14	Some post-secondary
ALS-4	67	M	2	4	College degree
ALS-5	69	F	11	23	College degree
ALS-6	52	M	3	22	Some post-secondary
ALS-7	61	M	1	39	College degree
ALS-8	54	F	2	41	Some post-secondary
ALS-9	67	M	2	26	Some post-secondary

Mean ± SD	58.2 ± 12.9	–	4.9 ± 4.0	19.2 ± 15.0	–

### Data Acquisition

**FIGURE 1:**
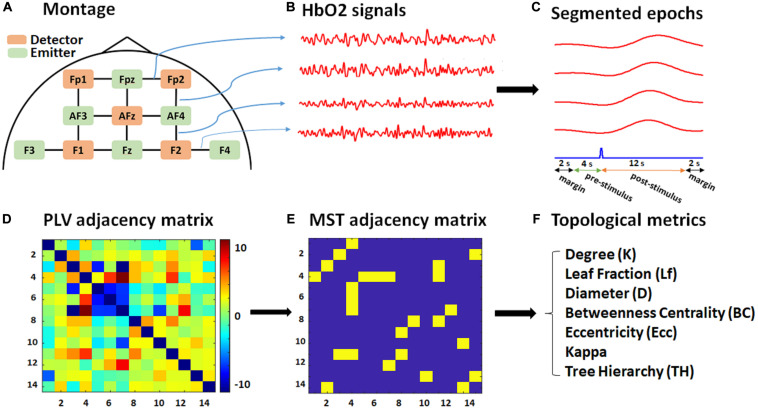
Schematic of the montage, data, and graph analysis. **(A)** The fNIRS recording montage, including detectors (orange), emitters (green), and 14 constructed channels (black). **(B)** Exported HbO2 time series for each channel (four of which are shown for illustrative purposes only). **(C)** Segmented 20 s epochs (red) corresponding to each channel with respect to a target stimulus (blue) consisting of 12 s post-stimulus, 4 s pre-stimulus, and 2-s margins at each edge. **(D)** PLV adjacency matrix (14 × 14) based on the average pairwise normalized PLV over the post-stimulus time for each subject. **(E)** MST adjacency matrix (14 × 14) extracted from the PLV adjacency matrix through the Kruskal algorithm. **(F)** List of topological metrics computed from the MST analysis.

Functional near-infrared spectroscopy data were recorded using the NIRScout system (NIRx Inc.) with two near-infrared wavelengths (760 nm and 850 nm) and digitized at a sampling rate of 7.81 Hz. Figure 1A shows the optode placements and channel configuration. We used six emitters (green) and five detectors (orange) constituting 14 channels over the pre/frontal cortical areas, commonly used in fNIRS studies for a variety of mental tasks, including mathematical operations (Bauernfeind et al., 2011; Schudlo and Chau, 2014). The emitters were located at Fpz, AF3, AF4, F3, Fz, F4, while the detectors were placed at Fp1, Fp2, AFz, F1, and F2, according to the modified combinatorial nomenclature (MCN) positioning system. A calibration test was performed prior to each recording to assess signal quality for each channel separately.

### Experimental Protocol

All subjects first participated in a familiarization session to be trained on the experimental protocol and then participated in a main experimental session. In this study, we used a novel visuo-mental (VM) paradigm as our experimental protocol, following our previous works, which showed its efficacy to evoke hemodynamic responses in ALS patients as well as healthy participants (Borgheai et al., 2020, 2019). The VM paradigm incorporates mental calculation alongside commonly used visual stimulation in brain–computer interface (BCI) communication systems. This augmentation of mental arithmetic has been reported to compensate for ALS patient’s incompetence in performing visual tasks, particularly in the later stages of the disease (Borgheai et al., 2020). Subjects were exposed to visual stimuli through a 23″ LCD monitor. For ALS patients, a holder kept the display before them at their bedsides. The VM stimulation paradigm was designed and presented through BCI2000 software (Schalk et al., 2004).

Participants each had two successive runs of the VM task in the main experimental session. In each run, participants were instructed to perform mental calculations using the 2 × 2 matrix of numbers intensified over the target character. The calculation included a simple addition/subtraction either diagonally (at the first flash) or vertically (at the second flash) within the intensified matrix, picking the greater value and multiplying it by two, as explained in more detail in our previous works (Borgheai et al., 2020, 2019). The stimulation time was set to 300 ms, and the inter-stimulation interval (ISI) was set to 6 s. In total, for two runs of the VM paradigm, there were 28 target characters (14 for each run), with one row/column flashes (single-trial) for each target character.

### Data Analysis

#### Signal Preprocessing

The modified Beer-Lambert law was used to calculate concentration changes for oxygenated hemoglobin (HbO2) and deoxygenated hemoglobin (HbR) in terms of recorded alterations in reflected light attenuation (see Figure 1B). fNIRS data were then band-pass filtered at 0.01–0.15 Hz to mitigate physiological noises caused by respiratory (∼0.2–0.3 Hz) (Barrett et al., 2016) and cardiac activities (∼0.8–2 Hz) (Chaddad et al., 2013), and to remove high-frequency noise (above 2 Hz). The exported data were then segmented into [−6 to 14] sec epochs relative to target stimulus onset. The total length of each epoch for phase analysis was therefore 20 s, consisting of 12 (post-stimulus) + 4 (pre-stimulus) + 2 × 2 (margins) (see Figure 1C). Including the 4 s pre-stimulus further supported baseline normalization for the connectivity analysis, in which the baseline period was selected from 4 to 1 s prior to target stimulus onset. The extra 2-s margin periods were selected to cancel edge effects in the later phase analysis and included at the beginning and ending of each epoch.

As HbO2 signals have been reported to reflect stronger effects in fNIRS connectivity analysis (Li and Yu, 2016) and also are more sensitive to the cerebral vascular changes than HbR signals (Vourkas et al., 2014), the HbO2 epochs were selected for further connectivity analysis.

#### Functional Connectivity

##### Phase-locking value (PLV) analysis

The Hilbert transform was used to calculate the instantaneous phase (φ[*n*]) for each epoch (*x*[*n*]). The Hilbert transform of *x*[*n*] in the frequency domain (*X*_*H*_) is defined as (Oppenheim and Schafer, 1998):

(1) $$X_{H}\left(e^{j\omega }\right)=-j\times sgn\left(\omega \right)\times X\left(e^{j\omega }\right)$$

where *X*(*e*^*j*ω^) is the Fourier transform of *x*[*n*] and *sgn*(ω) is the sign function with a value of +1 and −1 for positive and negative frequencies, respectively. Then, by calculating the inverse Fourier transform of *X*_*H*_ as *x*_*h*_, we constituted the analytical signal *x*_*a*_[*n*] as below:

(2) $$x_{a}\left[n\right]=x\left[n\right]+jx_{h}\left[n\right]$$

The instantaneous phase φ [n]was then calculated as:

(3) $$\varphi \left[n\right]=arctan\left(x_{h}\left[n\right]/x\left[n\right]\right)$$

Following similar fNIRS-based connectivity studies (Molavi et al., 2014; Li et al., 2018), the functional connectivity metric used in this study was the PLV (Shahriari et al., 2016; Cohen, 2019) that quantifies the synchronization level between the phases of two signals. For each pair of channels {*k*,*l*}, each time point (*n*), *PLV*_*kl*_[*n*] was calculated by averaging the instantaneous phase differences between the two channels over all the trials (*N*) as follows:

(4) $$PLV_{kl}\left[n\right]=\left|\frac{1}{N}\sum_{p=1}^{N}\exp\left(j\left(\varphi_{k}\left[p,n\right]-\varphi_{l}\left[p,n\right]\right)\right)\right|$$

where φ_*k*_[*p*,*n*] is the instantaneous phase of the signal in channel *k*, trial *p*, and time-stamp *n*. Note that *N*, here, is 28, i.e., the total number of target trials (epochs) for each session.

To extract only the task-related connectivity, the pairwise PLV index was then normalized with respect to the baseline period (−4 to −1 s), which is expected to cancel the stationary phase synchronization unrelated to the task (Doesburg et al., 2008; Mylonas et al., 2016). To carry out this normalization, we subtracted the mean PLV value of the baseline period from each pairwise PLV and then divided the result by the standard deviation of the same baseline. The normalized PLV (zPLV) calculation was formulated as below:

(5) $$zPLV_{kl}\left[n\right]=\frac{PLV_{kl}\left[n\right]-\mu_{kl}}{\sigma_{kl}}$$

where μ_*kl*_ and σ_*kl*_ are, respectively, the mean and standard deviation of the PLV in the baseline period between channels *k* and *l*. The use of normalized phase synchronization measures, i.e., zPLV, as the functional connectivity metric in our study, also makes the analysis less biased to the level of hemodynamic responses, which can vary across different subjects, performances, and recording environments (Handwerker et al., 2004; Hu et al., 2013).

### Graph Analysis

The pairwise normalized PLV index was used to construct weighted graphs, extract the topological properties, and then, accordingly, investigate between-group topological differences in the graphs’ characteristics. Figures 1D–F demonstrates the schematic pipeline for the graph analysis performed for each subject.

#### PLV Adjacency Matrix

To construct the PLV adjacency matrix, the normalized PLV (zPLV) index was averaged over all post-stimulus time-points for each channel pair, i.e., over [0–12] sec after the target stimulus onset. The total pairwise phase-locking value (tPLV) was formulated as below:

(6) $$tPLV_{kl}=\frac{1}{M}\sum_{p=1}^{M}zPLV_{kl}\left[p\right]$$

where *M* is the total number of samples for the 12-sec post-stimulus period (here *M* = 94 ∼ 12 (sec) × 7.81 (*sampling rate*). Accordingly, for every subject, all pairwise combinations of tPLVs resulted in a square PLV adjacency matrix (14 × 14), with the number of channels (14) as its dimension.

#### MST Adjacency Matrix

For each session and each participant, an undirected weighted graph was constructed by assigning each pairwise tPLV to the link (edge) between the corresponding channel (node) pair, as its weight. Kruskal’s algorithm (Kruskal, 1956) was then applied to the graph to obtain its MST. The MST network is a unique sub-graph that traverses all nodes by minimizing cost (edge distances) without forming a loop (Tewarie et al., 2015; van Dellen et al., 2018). In this work, following other connectivity-based MST network studies (Olde Dubbelink et al., 2014; Fraga González et al., 2016), the maximum connectivity tree was derived from each PLV adjacency matrix, mathematically equivalent to the MST constructed by Kruskal’s algorithm. In brief, we first sorted the links, in ascending order, with respect to their tPLV values. Then, starting with the edge with the highest tPLV, we continued assigning an unweighted edge to the tree, unless adding the link formed a cycle, where we skipped that edge and selected the next link in the sorted array. To detect cycles, we implemented the deep-first search (DFS) algorithm (Even, 2012), in which for every visited node ‘a,’ if there was an adjacent node ‘b’ that was already visited and was not the parent of ‘a,’ then a cycle was formed in the graph. A visited node is a node we have traversed before, and the parent node is the node for which the algorithm is currently tracing adjacent nodes. The final MST is thus an undirected unweighted graph, including all *N* nodes (i.e., 14) with *N* − 1 links (i.e., 13). Equivalently, the resulting MST adjacency matrix would be a binary matrix with values either 1 or 0 for a connected or disconnected node pair, respectively.

#### Topological Analysis

The topology of the extracted MSTs was characterized using network metrics obtained from the MST adjacency matrix analysis. These metrics were categorized into two main groups: global properties and local (nodal and edge) characteristics. The former provides global measures to compare networks as a whole, while the latter includes individual measures of relative nodal importance (Li et al., 2017; van Dellen et al., 2018). We compared network metrics (both global and local) between ALS and HC subjects at both the group and individual levels (i.e., group MST analysis and individual MST analysis). In the group MST analysis, we constructed the MST and its associated properties from the average PLV matrix over the subjects in each group, while in the individual MST analysis, first, we constructed individual MSTs from the individual PLV matrices of each subject in each group, and then we extracted network metrics from each individual MST. Before describing the topological metrics, we define the following basic terms: (a) degree (*k*), the number of edges connected to a node; (b) leaf node, a node with degree one, i.e., with a single connecting edge; (c) hub node, with multiple connected edges and, therefore, a degree greater than one. Two extreme networks are generally considered to investigate tree properties: (1) line-like tree (snake), which is a line traversing all the nodes. All nodes in a line-like network have degree two, except two starting and ending nodes with degree one, i.e., leaf nodes. (2) Star-like tree, in which one node is in the center of the network, connected to all other nodes. The central hub node has degree *N* − 1, where *N* is the total number of nodes in the network, and all other nodes have degree one. Below several network metrics used in this study are explained:

##### Local (node and edge) properties

The nodal measures used in this study are as follows: (1) degree (*k*). (2) Betweenness centrality (*BC*), a measure of how often each node sits on the shortest path between two other nodes. The shortest-path for each nodal pair is the minimum path between them. The BC for a node *r* is defined as below (van Dellen et al., 2018):

(7) $$BC\left(r\right)=\frac{1}{\left(N-1\right)\left(N-2\right)}\sum_{p,q\neq r}\frac{n_{pq}\left(r\right)}{N_{pq}}$$

where *n*_*pq*_(*r*) is the number of shortest paths between nodes *p* and *q*, which pass through the node *r*. *N*_*pq*_ represents the total number of shortest paths between nodes *p* and *q.* BC is 0 for a leaf node and is 1 for a central node in a star-like tree. (3) Eccentricity (*Ecc*), which represents node centrality and is the length of the longest ‘shortest-path’ from a reference node to any other node in a network. Eccentricity takes its minimum possible value of one for a central node in a star-like tree.

As in the group analysis, the individual characteristics of nodes and edges could not be completely revealed (de Reus and van den Heuvel, 2013), we further investigated the occurrence of edges and hubs among individual MSTs from subjects in each group. In this way, individual occurrence information complements the outcomes of group analysis.

##### Global properties

The global MST network metrics demonstrate the characteristics of the entire network across the brain. The global measures we used here include (1) leaf fraction (*L*_*f*_), which is the number of nodes with only one connected edge (*k* = 1), i.e., the number of leaf nodes (*L*), divided by the maximum possible number of leaves for a graph with *N* nodes, i.e., *N* − 1. For a star-like network, the leaf fraction is 1, while for a line-like network, it would be 2/(*N* − 1). (2) Maximum degree (*k*_*max*_), which is the maximum degree of a node in a network. (3) Maximum *BC* (*BC*_*max*_), which is the maximum *BC* value among the nodes of the network. (4) The *Ecc* of a whole MST network, which is defined as the difference between the largest and the smallest eccentricity values among the nodes in the tree. (5) Kappa or degree divergence, which is the measure of the variance of the degree distribution and is calculated as below (Tewarie et al., 2015):

(8) $$Kappa=\frac{\sum_{i}k_{i}^{2}}{\sum_{i}k_{i}}$$

where *k*_*i*_ is the degree associated with the node *i*. (6) Diameter (*D*), which is defined as the length of the longest shortest-path, normalized by (*N −* 1). The greater the diameter, the less central the network. (7) Tree hierarchy (*T*_*H*_), which characterizes a hypothesized optimal topology of an efficient organization while preventing information overload of central nodes. It can be interpreted as the balance between hub overload prevention and large-scale integration (Fraga González et al., 2016). *T*_*H*_ ranges between 0 and 1 and is calculated as below:

(9) $$T_{H}=\frac{L}{2\times \left(N-1\right)\times BC_{max}}$$

For a line-like network, *T*_*H*_ tends to 0, while in a star-like tree, it is 0.5 (*L* = *N* − 1).

(8) Similarity analysis, in which the fraction of edge overlap between individual MSTs and the reference MST (MSTref) was compared for both ALS patients and healthy controls. The MSTref was constructed from the average connectivity matrix of all healthy controls (Fraschini et al., 2016; van Dellen et al., 2018).

In addition to network topological properties, we calculated the global PLV (gPLV) for each individual subject by averaging the tPLVs over all elements (pairwise tPLV) of the PLV adjacency matrix for further group-comparison analysis.

### Statistical Analysis

The statistical significance of between-group differences for each global network metric was evaluated with the non-parametric Mann–Whitney *U* test (Wilcoxon rank-sum test). Moreover, for the ALS cohort, the Spearman correlation analysis was conducted to explore relationships between the global properties, as described above, and the ALS clinical scores, including disease duration and disability score (ALSFRS-R).

## Results

### Functional Connectivity

The global PLVs (gPLVs) that were calculated by averaging the PLVs over all channel pairs for each subject within each group were given to Wilcoxon rank sum statistical analysis. No significant between-group differences were observed for the gPLV (*p*-value = 0.447, see Table 2).

**TABLE 2:** Means (M) and standard deviations (SD) for the global network metrics in ALS and HC groups.

	*HC*		*ALS*		
	*M*	*SD*	*M*	*SD*	*p*-Value
Global PLV (*gPLV*)	1.04	1.85	0.18	3.76	0.447
Maximum degree (*K*_*max*_)	4.80	0.63	6.22	1.56	0.030
Leaf fraction (*L*_*f*_)	0.58	0.05	0.68	0.08	0.029
Diameter (*D*)	0.49	0.04	0.41	0.09	0.040
*Maximum BC (BC_*max*_)*	0.36	0.04	0.39	0.05	0.117
Eccentricity (*Ecc*)	3.00	0.00	2.33	0.50	0.006
Kappa	2.59	0.17	3.12	0.49	0.005
Tree hierarchy (*T*_*H*_)	0.82	0.12	0.89	0.15	0.325
Overlap (%)	30.77	12.03	19.66	10.96	0.051

### Group MST Comparison

**FIGURE 2:**
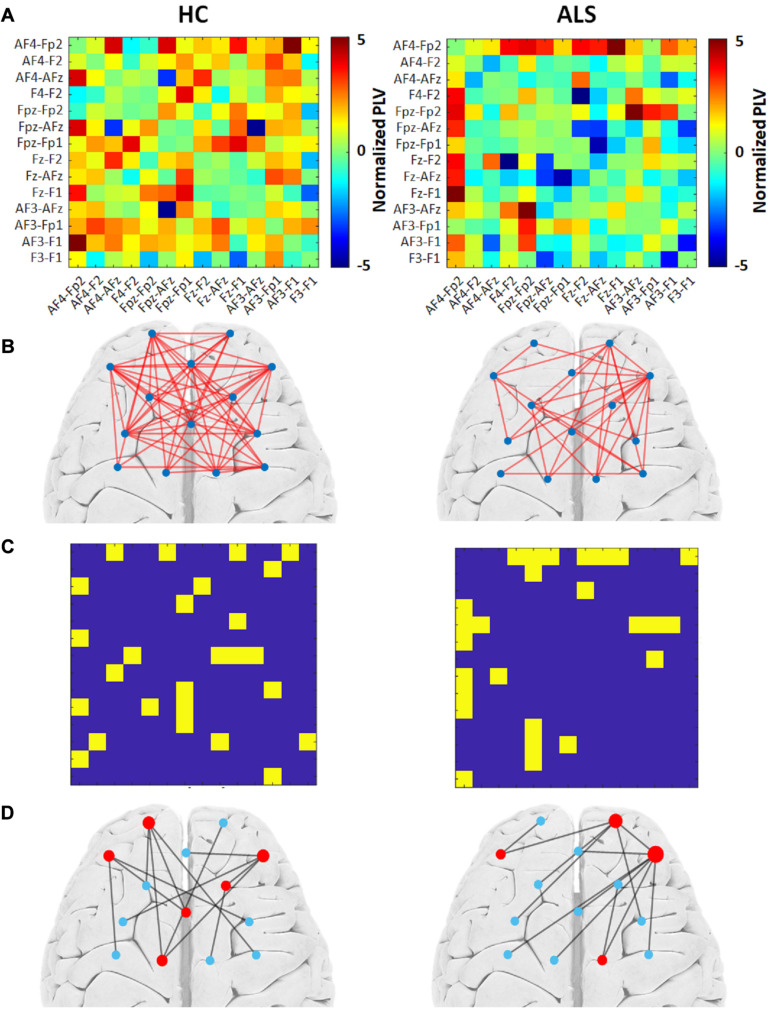
The group MST graph analysis for both the healthy control (HC) (left) and ALS (right) groups. **(A)** Average PLV adjacency matrices over all subjects in the group. **(B)** The weighted graphs constructed based on group average PLVs with an arbitrary threshold of 15% of the highest value. **(C)** Unweighted MST adjacency matrices extracted through the Kruskal algorithm. **(D)** Corresponding MST graphs. The radius of each node represents its degree. Leaf nodes (degree = 1) are blue, while hub nodes (degree > 1) are red.

Figure 2 illustrates the outcomes of the graph analysis for each group (HC and ALS). Specifically, Figure 2A shows the PLV adjacency matrices constituted from the average normalized PLVs over all subjects in each group. Figure 2B shows the weighted undirected graphs constructed based on the PLV adjacency matrices in each group. For visualization purposes only, we have shown the links with weights greater than 15% of the maximum normalized PLVs in each group. This threshold was arbitrarily selected to project a general schematic of the corresponding weighted graphs. By applying the Kruskal algorithm to these weighted graphs, unweighted (binary) MST adjacency matrices were extracted from each group (Figure 2C). Corresponding MST graphs, which will henceforth be called ‘group MST,’ were projected over a cortical brain area in Figure 2D. The radius of each node represents its degree, i.e., the numbers of edges connected to that node. Leaf nodes (degree = 1) are blue, while hub nodes (degree > 1) are red. In the group MST for ALS group, we observed the highest degree at channels AF4-Fp2 and Fpz-Fp2 with degrees 7 and 5, respectively, i.e., *Kmax* = 7. In the group MST for healthy controls, the nodes with the highest degree were observed in the channels AF4-Fp2 and Fpz-Fp1, both with degree 4, i.e., *Kmax* = 4. *TH* and *BCmax* in the group MST for ALS were 0.97 and 0.40, respectively, compared to respective values of 0.94 and 0.33 in the group MST for controls. The comparatively higher values of *Kmax* and *BCmax* in the group MST for ALS are associated with more load on the corresponding nodes compared to controls. The group MST for ALS included ten leaf nodes (*Lf* = 0.77), compared to eight leaf nodes (*Lf* = 0.62) in the group MST for healthy controls, which indicates more centralization in the ALS cohort’s group MST. The diameter and global eccentricity in the group MST for ALS were 0.38 and 2, respectively, compared to respective values of 0.54 and 3 in the controls’ group MST. These metrics provide additive evidence of a more centralized network in the group MST for ALS than for controls. Furthermore, the six hubs of the group MST for controls were distributed more evenly over both pre/frontal hemispheres, while three of four hubs in the group MST for ALS resided in the right pre/frontal hemisphere. The group MST for ALS had only three edges (i.e., 23.08% of the edges in the ALS group) in common with the group MST for controls, which was later used as the reference for the dissimilarity analysis (MSTref).

### Individual MST Analysis

#### Comparison of Global Metrics

**FIGURE 3:**
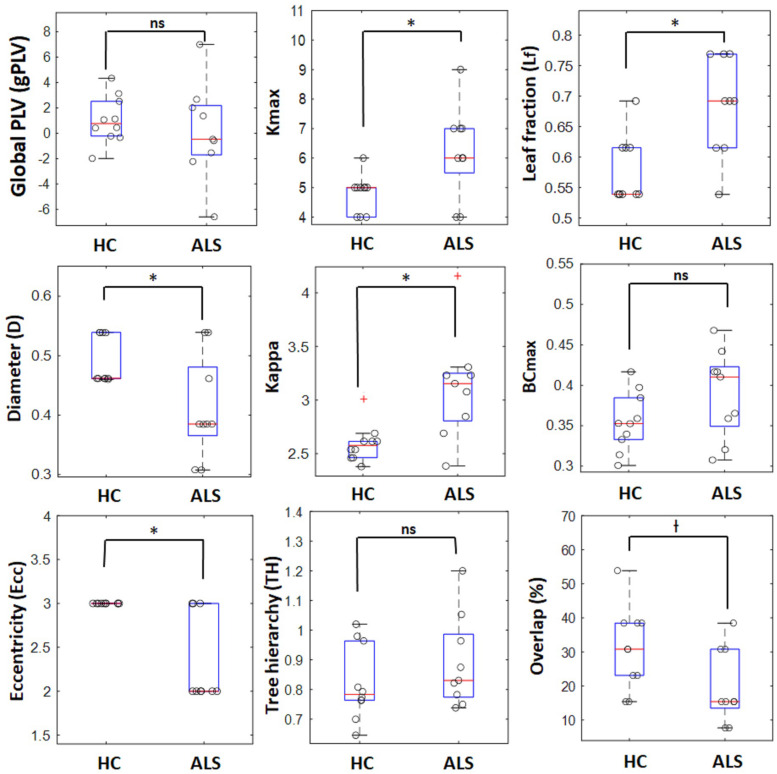
Boxplots for global network properties in both groups. In each box, the central red line denotes the median value, and the lower and upper limit of the blue box, respectively, denote the 25 and 75 percentiles. The outliers are shown with red plus signs. o : represents data points for individual ALS patients and healthy controls, ^∗^ significant (*p*-value < 0.05), ^†^ marginally significant (*p*-value ∼ 0.05), ns, non-significant.

The box plots in Figure 3 shows how the global metrics for individual MSTs were distributed in both the ALS and HC groups and Table 2 lists the corresponding means and standard deviations of the global metrics obtained from individuals in both cohorts. The non-parametric Mann–Whitney *U* test revealed significant differences between ALS and HC for the majority of the global MST topological metrics. Specifically, we observed significantly higher values of maximum degrees (*Kmax, p*-value = 0.030) and leaf fractions/numbers (*Lf, p*-value = 0.029) in the ALS cohort compared to controls. Complementary to the group analysis, these results show more centralized global networks in the patient group. The Kappa value was also significantly (*p*-value = 0.005) lower in controls, which indicates less variability in the degree distribution over the nodes in individual MSTs for healthy subjects. The diameter (*D*, *p*-value = 0.040) and global eccentricity (*Ecc*, *p*-value = 0.006) were significantly higher in healthy controls than in patients. Overall, all global network measures, except for overlap, showed higher standard deviations in patients than in controls, indicating less variability and more robust networks in the controls. Additionally, similarity analysis showed marginally significant higher overlap (*p*-value = 0.051) in the individual MSTs of controls compared to the patients, as an additive support for more consistent topological variation over the healthy cohort.

#### Comparison of Local Properties

**FIGURE 4:**
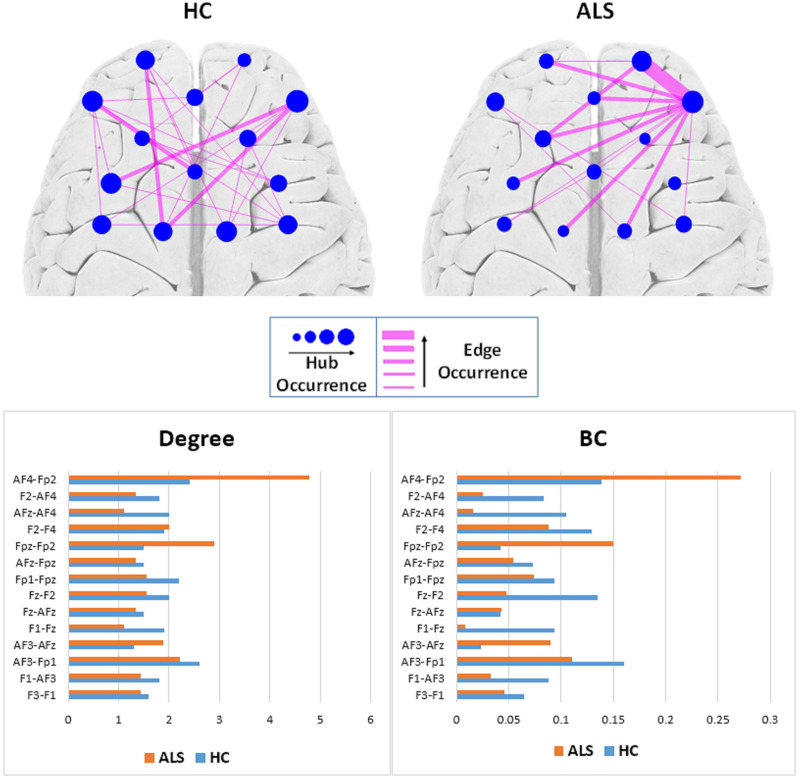
Top: Comparison of hub and edge occurrences between the two groups of ALS and healthy control (HC). Bottom: Nodal average degree (left). Nodal average betweenness centrality (BC) values (right).

Figure 4 (top) shows the occurrence of edges and hubs in individual MSTs for the ALS and HC subjects. Nodes, which appear more frequently as hubs for subjects in that group, are shown as circles with larger radii, while less frequently occurring hubs have smaller radii. Similarly, thicker edges indicate the more frequent presence of that edge in individual MSTs for that group. The most frequent MST hubs in ALS subjects were channels AF4-Fp2 and Fpz-Fp2, with respective occurrences of 77.8% and 66.7%. In healthy controls, the hubs were distributed more evenly over the frontal cortex, with the most frequent hubs appearing at channels AF4-Fp2 with 70.0% and Fz-F2, AF3-F1, and AF3-Fp1 with 60.0% occurrence.

To remove visual complexity, the edges shown are limited to those that occur in more than 25% of the subjects in each group. In the ALS cohort, the most commonly occurring edge was in the right prefrontal area, between channels Fpz-Fp2 and AF4-Fp2, appearing in 66.7% of the patients’ MSTs, i.e., 6 out of 9. In the healthy controls, we observed a more even distribution of edge occurrence over the frontal cortex with most commonly occurring edges connecting channels AF3-Fp1 and Fz-AFz, channels Fpz-Fp1 and Fz-F1, channels AF3-F1 and AF4-Fp2, and channels Fz-F1 and AF4-Fp2 in 40% of the MSTs of healthy controls, i.e., 4 out of 10.

Figure 4 (bottom) illustrates the average degree and *BC* of each node (channel) over all the subjects in each group. In the ALS cohort, we observed the highest average degree and *BC* at channel AF4-Fp2, in the right prefrontal cortex, with respective values of 4.78 ± 3.03 and 0.27 ± 0.19. In the HC group, we observed the highest average degree and *BC* at channel AF3-Fp1, in the left prefrontal cortex, with 2.60 ± 1.78 and 0.16 ± 0.18, respectively.

**FIGURE 5:**
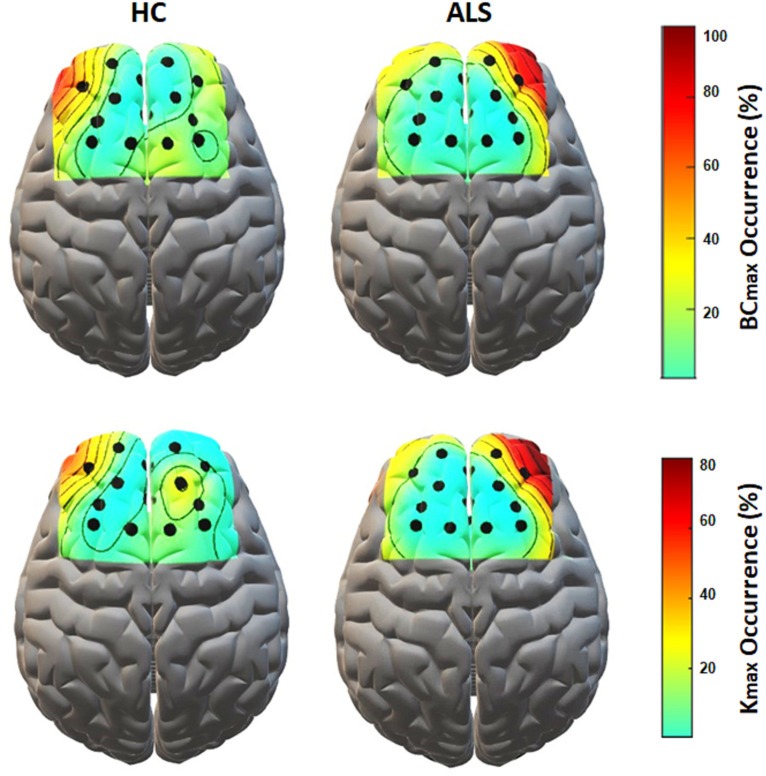
The topological mapping of nodal occurrence of maximum BC (top) and maximum degree (bottom) in each node in each group. The maps are superimposed on a standard 3D brain model for illustrative purposes only. Dots show the corresponding fNIRS channel over the cortex.

Figure 5 shows the topological representation of the nodes with the occurrence of maximum *BC* (*BCmax*) and degree (*Kmax*) in individual MSTs for both groups. In the ALS group, both *BCmax* and *Kmax* occurred most frequently at channel AF4-Fp2, in the right prefrontal cortex, in 55.6% of the subjects for each. However, in the controls, the highest occurrence of *BCmax* and *Kmax* was at channel AF3-Fp1, in the left prefrontal cortex, in 44.4% and 33.3% of the subjects, respectively.

### Associations Between Global Network Properties and Clinical Data

**FIGURE 6:**
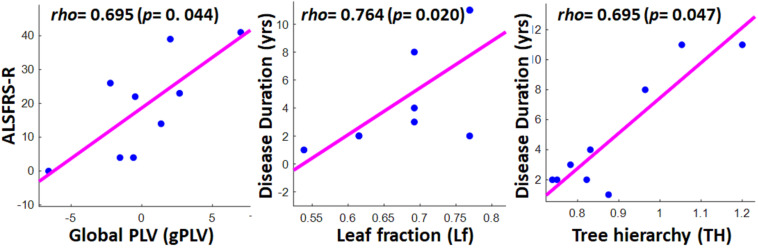
Scatter plots showing the significant correlations between clinical scores in ALS group and global network properties, including global PLV (left), leaf fraction (middle), and tree hierarchy (right). The respective correlation’s *rho* and *p*-value are represented on the top left of each plot.

Figure 6 illustrates the significant results derived from the Spearman correlation analysis between the global network properties and clinical scores in the ALS group. Although no significant correlation was observed between global MST metrics and disability score (ALSFRS-R) in the ALS cohort, we observed a significant correlation between global PLVs (gPLVs) and disability scores (*p*-value = 0.044). Moreover, among the network metrics, leaf fraction (*Lf*, *p*-value = 0.020) and tree hierarchy (*TH, p*-value = 0.047) showed significant correlations with disease duration.

## Discussion

In this study, we applied MST network analysis to fNIRS-based hemodynamic responses to a visuo-mental task to compare frontal functional brain network topology between ALS patients and healthy controls and further link the outcomes to executive dysfunctions reported across ALS studies. We used PLV-based phase synchronization connectivity measures to calculate functional inter-channel relations over which the individual and group MSTs were constructed. This study also investigated how patients’ clinical scores are associated with their global connectivity and topological metrics. The MST network analysis employed in the present study revealed both global and local disruptions in frontal network properties in ALS patients in relation to controls. Compared to more commonly used network analysis methods, MST has been shown to be less influenced by spurious connectivity due to its intrinsic dimensionality reduction of the links (Tewarie et al., 2015). So, the construction of the MST results in a unique and more robust network representation of brain network organization and captures the core topological properties essential for a less-biased comparison of brain network organizations across different groups, conditions, and studies (van Wijk et al., 2010; Tewarie et al., 2015).

Globally, both group and individual MST analyses suggested a shift toward a more centralized frontal network organization for the ALS patients compared to the controls. A more centralized network is, in general, a more star-like configuration, which has been interpreted as more random or dysregulated as opposed to a more regular network (Fraga González et al., 2016; Li et al., 2017). Tending to a more random structure in the ALS group is an indicator of lower clustering and a shorter path length (Tewarie et al., 2015), which is aligned with the observed smaller diameter and higher leaf fraction in this cohort than in the HCs. The higher centralization in the more star-like patient MSTs maintains that the information traverses along fewer limited and overloaded nodes that disrupts the quality of information exchange. This is also an indicator of the tendency toward a more ‘scale-free’ type of network (Stam, 2014; Otte et al., 2015), which similarly denotes the existence of hubs with a high density of connections (degree). This is corroborated by the higher maximum degree and betweenness in the patients’ cohort, meaning that most information is exchanged between only a few central hubs. In addition, aligned with other metrics of centralizations, higher values of kappa (degree divergence) in patients suggest the existence of high-degree nodes, causing a more rapid synchronization, i.e., linking more likely to the higher density nodes, and at the same time making the network more vulnerable to noise (Otte et al., 2015). Our observed global pattern of a more centralized network organization in patient MSTs is aligned with the findings reported by Sorrentino et al. (2018) in a recent MEG–based MST network analysis study on a cohort of ALS patients that reported a shift toward a more centralized brain network in all frequency bands as the disease progressed. This shift toward a more centralized and random network has also been reported in MST-based studies on other neurological diseases such as schizophrenia (Jonak et al., 2019) and major depressive disorder (MDD) (Li et al., 2017). Topological disruption in the global MST networks of ALS patients compared to healthy controls has also been reported by Fraschini et al. (2016) in an EEG-based RSFC study, although it was not consistent with the aforementioned centralization trend within the beta-band. The inconsistency in the global beta-band network characteristics in the previous work might be attributed to the local topological difference related to motor functions associated with ALS (Kasahara et al., 2012; Proudfoot et al., 2017). The inconsistency may also be related to a possible difference in the clinical and cognitive characteristics of the patient population (Fraschini et al., 2016) and/or methodological sensitivity to the selection of regions of interest (ROI) (Sorrentino et al., 2018). Our work differs from the previously reported MST-based studies on ALS as first, our paradigm is activity-based in contrast to the previous resting-state studies, and second our study is frontally focused, unlike previous widespread reports on whole brain organization. Thus, considering the conflicting trends reported in MST-based global network analyses in ALS, integrating further local and nodal network analysis in the frontal area of the cortex might lead to new findings on cognitive aspects to compensate for the inconsistency and play a complementary role for the existing motor-related network findings.

Our nodal analysis characterized the main local hubs, which are highly connected nodes that serve as relay stations and are responsible for transferring information across dispersed brain modules for each group (Bullmore and Sporns, 2012). The importance of identifying the hubs here stems from their critical contribution to cognitive processes (Sheffield et al., 2015) and performance (Crossley et al., 2013). These hubs are reported as common targets of neurodegenerative conditions (Gleichgerrcht et al., 2015). Here, in the healthy controls, hubs are distributed more evenly over the frontal cortex with a slightly higher occurrence in the left prefrontal cortex (PFC), while in the patients, the most frequent hubs were asymmetrically observed, particularly in the right PFC. The high-density nodes in the right PFC and the highest occurrence of maximum degree and betweenness centrality jointly identified these nodes as central hubs in the ALS cohort. Overall, compared to controls, it can be implied that efficiency and node strength in ALS patients decreased in the left PFC area and increased in the right PFC area. This lateralized processing in the prefrontal cortex is in accord with what has been reported in a positron emission tomography (PET) neuroimaging ALS study by Abrahams et al. (1996) with a verbal fluency task and an event-related potential (ERP) study by Hammer et al. (2011), where ALS patients performed a dual-task constituted from a spatial and a WM n-back task. These two studies linked PFC-related deficits in ALS to specific task-related executive dysfunctions in these cohorts, which, rather than merely requiring passive storage of data, actively engaged WM to manipulate incoming information. This supports our current observation of the PFC alteration, as our proposed dual-task required active manipulation of numbers in the mental arithmetic component of the task, which has been broadly linked to WM processing (Dehaene et al., 2004; Klein et al., 2013) and particularly associated with hemodynamic activity in the PFC (Sammer et al., 2007; Schudlo and Chau, 2014). This also aligns with our previous findings (Borgheai et al., 2019), where we observed a significant contrast, primarily in the PFC region, in ALS patients compared to the controls, using a simple first-order feature (i.e., the integral of hemodynamic activities) in response to the same paradigm. One explanation for this DLPFC asymmetric processing and general frontal topological alteration is “task-specific” executive dysfunction in the ALS cohort, in particular deficits in task-related WM processing. This interpretation is in accord with other behavioral studies reporting executive dysfunctions in ALS, particularly WM and fluency impairments (Frank et al., 1997; Abrahams et al., 2000). Specifically, as the right PFC is suggested to contribute to executive control, including inhibitory functions (Monden et al., 2015) and memory retrieval monitoring (Henson et al., 1999), our derived high-density hubs in the right prefrontal lobe in ALS patients might be attributed to excessive compensatory executive attempts in these cohorts to maintain control over task-related WM processing. Moreover, as the right prefrontal cortex has also been reported to be involved in spatial WM tasks (Causse et al., 2017), our observed right PFC hyper-connectivity in the ALS group can be attributed to their dysfunctions in handling the spatial aspect of our tested dual task. This frontal lateralization has also been reported by Hammer et al. (2011) in ALS patients, as well as other neurological diseases including schizophrenia (Glahn et al., 2005) and MDD (Li et al., 2017), with higher activation in the right frontal lobe. Similarity with executive neuro-topological markers of the depressive disorder has an additive explanatory value, as there is a consistent body of literature associating depressive symptoms with ALS (Wicks et al., 2007; Roos et al., 2016; Thakore and Pioro, 2016). Another explanation for the observed frontal network alterations in ALS is the plausible attribution of functional dysfunction to reported ‘ALS-specific’ structural atrophies, mainly reported in ‘task-negative’ RSFC studies (Douaud et al., 2011; Dimond et al., 2017). For example, in a network-based resting-state MRI-DTI study, Dimond et al. (2017) concluded that executive dysfunction in ALS patients is related to reduced white matter integrity and associated with deviations in global and local network properties with a high frontal and temporal preference. Specifically, they report associations between behavioral verbal fluency errors and clustering coefficient alterations, primarily in the right frontal and temporal lobes. Similarly, Tedeschi et al. (2012) reported suppression in the right fronto-parietal network in ALS, possibly due to the patients’ frontal dysfunction and right-lateralized patterns of regional atrophy. However, the direction of connectivity alteration is not consistent across resting-state studies, reporting both increases and decreases in connectivity measures or associated network metrics (Douaud et al., 2011; Agosta et al., 2013). Nonetheless, we can only connect task-negative (resting) functional connectivity findings to our current activity-based paradigm in which structural alterations can affect the functional network, which can be consequently reflected in both task-negative and task-positive conditions. Accordingly, we can follow the interpretive line suggested by RSFC studies that cortical connectivity alterations, in particular hyper-excitability, are a mechanism to compensate for structural atrophies (Agosta et al., 2013). This hyper-connectedness has also been attributed to a progressive loss of inhibitory cortical neurons as part of ALS pathogenesis (Turner and Kiernan, 2012). Additionally, these local atrophies can underlie the dysfunction of task-related cognitive executive networks, including the executive control network (ECN), fronto-parietal network (FPN), and dorsal attention network (DAN), which all cover the frontal regions and are believed to contribute to a range of cognitive dysfunctions in ALS patients (Christidi et al., 2012; Tedeschi et al., 2012).

Although our obtained global PLV values (gPLV) did not show any significant between-group differences, we observed a significant association in patients between their gPLVs and their disability scores (ALSFRS-R). While two other EEG-MST and MEG-MST studies on the ALS cohorts (Fraschini et al., 2016; Sorrentino et al., 2018) did not report any significant association between clinical scores and their phase synchronization measure, i.e., phase lag indexes (PLI), here the global normalized PLVs derived from the hemodynamic responses showed a significant association with patients’ disability scores. This suggests that fNIRS-based gPLVs, as a global network characteristic, can, *per se*, be introduced as a potential neurological marker for ALS pathogenesis, even before any MST being derived from it. Furthermore, the marginally significant difference in the overlap of individual MSTs in each group with the MSTref, together with the less variability of topological metrics in the healthy group, implies that MSTs can potentially provide a clinically relevant reference graph to assess possible ALS pathogenesis. Among the topological properties extracted from the MSTs, leaf fraction, and tree hierarchy were positively correlated with the duration of disease. The former metric leads to the speculation that as the disease continues, the frontal network tends toward a more centralized and dysregulated organization. The latter association, i.e., tree hierarchy with disease duration, suggests that the longevity of the disease leads to a suboptimal balance between hub overload and network integration (Freeman, 1977). This is partly aligned with the association between tree hierarchy and clinical scores reported in the two aforementioned studies, though they reported associations with ALSFRS-R scores rather than disease duration. As in a broader sampling of the ALS patients, functional disability scores are reported to be associated with disease duration (Beghi et al., 2007), our reported association with disease duration and not with ALSFRS-R scores might be due to our relatively small sample size.

### Limitations and Future Work

One limitation of this study can be attributed to the probable information loss due to the intrinsic nature of the MST-based network analysis in ignoring lower ordered connections and the dimensionality reduction in the connectivity matrices. Although MST methods can capture core network properties and are less likely to be influenced by connectivity strength and network density, this possible information loss can consequently make some network properties more sensitive to the network size. This suggests that our findings should be replicated with a higher density of optodes distributed widely over the brain. Moreover, methodically, this work was limited to MST as an unweighted and undirected graph analysis to allow unbiased comparison of the topological outcomes and also, in general, to investigate the feasibility of adopting this approach for hemodynamic responses in ALS patients as opposed to healthy controls. Nonetheless, it would be informative to examine conventional weighted network analysis such as the clustering coefficient and the average shortest path length measures (Olde Dubbelink et al., 2014; Tewarie et al., 2015) to comparatively evaluate the efficacy of MST based approach employed here. A parallel investigation of the structural relevance of our findings together with more cognitively oriented behavioral batteries, such as the Edinburgh Cognitive and Behavioral ALS Screen (ECAS) or Cognitive and Behavioral Screen (CBS), can also help extend our findings to explore the underlying behavioral and structural executive dysfunctions reported sporadically in similar ALS studies. Moreover, the statistical power of our study was limited by its relatively small sample size due to the rare nature of the disease and the difficulty of conducting an activity-based paradigm with partly/completely locked-in ALS patients. For future work, replicating our results with larger sample sizes would facilitate the generalizability of the observed neuropathological and prognostic markers of the disease. The other limitation of our work was the gender gap between the two cohorts, which is partly influenced by the gender imbalance reported in the ALS patients (Cacabelos et al., 2016), and may have influenced our results. Thus, future works should consider the statistical effect of gender on our topological outcomes.

## Conclusion

In the present study, for the first time, we have demonstrated that MST analysis can mirror frontal changes in functional network topology in ALS patients during activity-based tasks. This frontally focused analysis can specifically reveal executive impairments associated with the disease and can generally be integrated with other ALS-targeted studies to play a complementary role with existing motor-related network findings. We also showed the feasibility and clinically relevant applicability of MST network analysis in fNIRS-based hemodynamic responses.

In summary, our analyses demonstrated a shift toward a more centralized and asymmetric frontal network organization in ALS cohorts compared to controls. Furthermore, it was demonstrated that the global PLV synchronization metric is associated with disease progression, and a few topological properties, including leaf fraction and tree hierarchy, are linked to disease duration. These findings suggest that hemodynamic-based network analysis during activity can possibly provide new neuro-topological markers for the bedside assessment of the functional status of ALS patients. Moreover, the methodologies developed in this study can be further extended to explore network disruption in other neurodegenerative diseases.

## Data Availability Statement

The data analyzed in this study is subject to the following licenses/restrictions: The data are restricted to be publicly available, as they contain confidential information that may conflict with the privacy of the research participants. Requests to access these datasets should be directed to yalda_shahriari@uri.edu.

## Ethics Statement

The studies involving human participants were reviewed and approved by Institutional Review Board (IRB) of the University of Rhode Island. The patients/participants/authorized witnesses provided their written informed consent to participate in this study.

## Author Contributions

YS had supervised all the aspects of this project, including the IRB process, patient and healthy control recruitment, data recording, data analysis, the interpretation of the results, and the manuscript preparation. SB had conducted the data recording, performed all the necessary computational analyses, interpretation of the results, and primarily completed the manuscript. JM had primarily assisted in the subjects’ recruitment, data recording, and proofreading of the manuscript. KM had assisted in fNIRS-related technical aspects of the study and proofreading of the manuscript. All authors contributed to the article and approved the submitted version.

## Conflict of Interest

The authors declare that the research was conducted in the absence of any commercial or financial relationships that could be construed as a potential conflict of interest.
